# Endoscopic Significance of Incidental Upper Gastrointestinal Wall Thickness Detected on Computed Tomography Scans

**DOI:** 10.7759/cureus.74609

**Published:** 2024-11-27

**Authors:** Zahid Idrees, Hashim T Khan, Usman Khan

**Affiliations:** 1 Gastroenterology and Hepatology, Lancashire Teaching Hospital, Preston, GBR; 2 Internal Medicine, Lancashire Teaching Hospital, Preston, GBR; 3 Internal Medicine, Royal Preston Hospital, Preston, GBR

**Keywords:** benign gi findings, endoscopy, esophageal cancer (ec), incidental gastrointestinal wall thickness, stomach cancer

## Abstract

Background

Incidental upper gastrointestinal wall thickness (GIWT) is a nonspecific finding often observed on computed tomography (CT) scans performed to investigate patients admitted with various symptoms both gastrointestinal (GI) and non-GI. Its clinical significance is uncertain, and patients often undergo endoscopic evaluation under an urgent cancer pathway to exclude malignancy. We aimed to conduct this study to determine how well the CT findings correlated endoscopically.

Methods

A retrospective data collection was performed for patients who were referred to the endoscopy department between July 2021 and July 2024 for evaluation of GIWT over a period of four years. We analyzed age, gender, reason for initial CT, endoscopic findings, histology findings, and evidence of cancer.

Results

Our final cohort included 50 patients who underwent endoscopic and subsequent histological examination following abnormal CT findings. The mean age of the included cohort was 71. Thirty-one were males and 19 patients were females. Ninety percent had esophageal wall thickness while 10% had gastric wall thickness. Twenty-six percent of patients had red flag symptoms such as weight loss and iron deficiency anemia as an indication for undergoing CT scans. About 8% of the patients were confirmed to have malignancy on endoscopy of which 6% had red flag symptoms such as weight loss and iron deficiency anemia. Seventy-eight percent of the cohort had gastroscopy within two to four weeks, 10% in four to 12 weeks and 12% after 12 weeks.

Conclusion

Our results illustrate that a majority of people with incidental GIWT had benign pathologies whereas a small number of patients had malignancy. It is worth noting that malignancy was more common among patients who had red flag symptoms, and therefore urgent endoscopy assessment under the urgent cancer pathway is recommended in these patients. On the contrary, the risk is noted to be low in patients with no red flag symptoms, therefore we recommend assessing such patients individually for the need for endoscopic evaluation based on detailed history and examination. We think further multicenter large studies are required in this area of research and develop standard protocols in relation to investigating incidental upper GIWT with endoscopy.

## Introduction

The use of imaging has made a significant change in our management of patients. However, increased use of imaging has also led to the discovery of findings that are unrelated to the clinical question asked by the clinician [1,2]. Incidental findings such as increased wall thickening of the stomach and esophagus found on computerized tomography (CT) scans push medical professionals to investigate further owing to the risk of esophageal and gastric cancers which are aggressive malignancies with poor prognoses due to their late detection [3]. In contrast, early detection of these cancers can mean curative endoscopic treatment [4]. However, increased gastrointestinal wall thickness (GIWT) on CT is a non-specific finding and can be due to a range of other benign gastrointestinal (GI) conditions such as hiatus hernia, esophagitis, gastritis, and candidiasis as well as edema caused by hypoalbuminemia [5,6]. Previous studies have advocated for using upper GI endoscopic evaluation with or without biopsy to investigate these scan findings - an invasive and costly procedure [7]. In the UK, these patients are usually referred via an urgent cancer referral pathway especially if they have what we have classified as “red flags” symptoms such as weight loss, iron deficiency anemia, dysphagia, or Malena [8]. To our knowledge, there are currently no guidelines regarding the triage of these referrals especially in the absence of symptoms, and few studies explore the diagnostic accuracy of CT scan findings when evaluated endoscopically. Our single-center study aims to add to this area of research by looking retrospectively at patients who had GIWT on CT scans and the results from the direct endoscopic evaluation of those findings.

## Materials and methods

This was a retrospective observational study conducted at two hospitals under Lancashire Teaching Hospitals NHS Foundation Trust between July 2021 and July 2024. A list of patients referred to the endoscopy department with indication-coded abnormal CT findings was retrieved by running a search through the electronic record (Flex, Sectra (Flex Ltd., Austin, TX), and endoscopy referral form) systems with software called Dbeaver (DBeaver Corporation, New York City, NY) and SQL Server Management Studio 18 (Microsoft, Redmond, Washington, DC). All patients with incidental esophageal and stomach wall thickness identified on CT scans who subsequently underwent endoscopic evaluation and histological examination were included in this study. The indications for undergoing initial CT imaging were diverse, encompassing both GI (nausea, vomiting, diarrhea, abdomen pain), non-GI (shortness of breath, chest pain, and chest infection), and red flag symptoms (weight loss, dysphagia, and iron deficiency anemia) in hospitalized patients. We reviewed the electronic records of 63 patients and thirteen patients were excluded based on the exclusion criteria - 1) malignancy confirmed on CT scan, 2) prior diagnosis of malignancy, 3) gastroscopy not performed in the best interest of patient due to frailty, and 4) patients who did not attend (DNA) their endoscopy appointment or lost to follow up with the department. Among excluded patients, three of them had confirmed malignancy on CT scan, three had a prior diagnosis of cancer, five lost follow-up or DNA, and two were not investigated due to frailty and multiple comorbidities. Therefore, our final cohort comprised 50 patients.

This study aimed to evaluate the clinical significance of incidental upper GIWT in relation to underlying pathologies, both malignant and benign, confirmed with macroscopic assessment by endoscopy and histological examination performed on biopsy samples. The second outcome was to determine the association of red flag symptoms with malignancy in this group of patients and the time from referral to endoscopy performed. Electronic records of patients were reviewed and data collected including metric data for patients, CT findings, indication for why the scan was performed, time from referral to endoscopy performed as well as the macroscopic finding of endoscopy and histological results of biopsies. Data analysis was performed by using Microsoft Excel (Microsoft, Redmond, Washington, DC).

## Results

A total of 50 patients were included in this study based on the inclusion criteria of patients with increased IUGIWT who underwent endoscopic examination. About 62% (n=31) of our cohort were men while 38% (n=19) were female. The mean age of patients was 71. In total, 86% (n=43) of our population had esophageal wall thickness (EWT). Of these patients, 48% (n=24) had lower EWT, 20% (n=10) had lower EWT extending to the gastroesophageal junction (GOJ), 10% (n=5) had mid-EWT and 8% (n=4) had GOJ thickening. Only 14% (n=7) of patients had gastric wall thickness. About 52% (n=26) of the population had a CT scan in order to investigate non-GI symptoms and were found to have IUGIWT, while 26% (n=13) had red flag symptoms and 22% (n=11) had GI symptoms including diarrhea, vomiting, dyspepsia, and abdomen pain.

Overall, 8% (n=4) of patients were confirmed to have malignancy on gastroscopy and histology, 12% (n=06) had Barrett’s esophagus, 58% (n=29) had hiatus hernia, esophagitis and gastritis, 4% (n=2) had candida esophagitis, 4% (n=2) had polyps, 2% (n=1) had esophageal stricture, 2% (n=1) had motility disorder, 2% (n=1) were Campylobacter-like organism (CLO) positive and 8% (n=4) had a normal endoscopy (Table 1).

**Table 1:** Endoscopic findings of patients with incidental upper wall thickness CLO = Campylobacter-like organism

Endoscopic findings	Proportion	Number of patients
Malignancy	8%	N=04
Barret’s esophagus	12%	N=06
Hiatus hernia, gastritis, and esophagitis	58%	N=29
Esophageal stricture	2%	1
Motility disorder	2%	1
Candida esophagitis	4%	2
Polyps	4%	2
CLO positive	2%	1
Normal	8%	4

All patients who were found to have cancer were men and the mean age was 70. On further analysis, 75% (n=3) had esophageal cancer and 25% (n=1) was diagnosed with stomach cancer. About 75% (n=3) of patients in the cancer group had red flag symptoms and 25% (n=1) were asymptomatic (Table 2). Three of the four patients had mural thickening while there was lower EWT in one patient.

**Table 2:** Detailed analysis of patients with malignancy and Barrett’s esophagus N = Number, RFS = Red Flag Symptoms, GIS = Gastrointestinal Symptoms, OAC = Esophageal Adenocarcinoma, DLBC = Diffuse Large B-cell Carcinoma.

Endoscopic findings	Number of patients	Indication for CT	Histology findings
Malignancy	N=4 (8%)	RFS N=3(75%), non-GIS N=1(25%)	N=3 (75%) OAC, 1 (25%) DLBC of stomach
Barret’s esophagus	N=06 (12%)	RFS N=3 (50%) and non-GIS N=3 (50%)	N=5 intestinal metaplasia, N=1 ulcer and granular tissue

**Figure 1:**
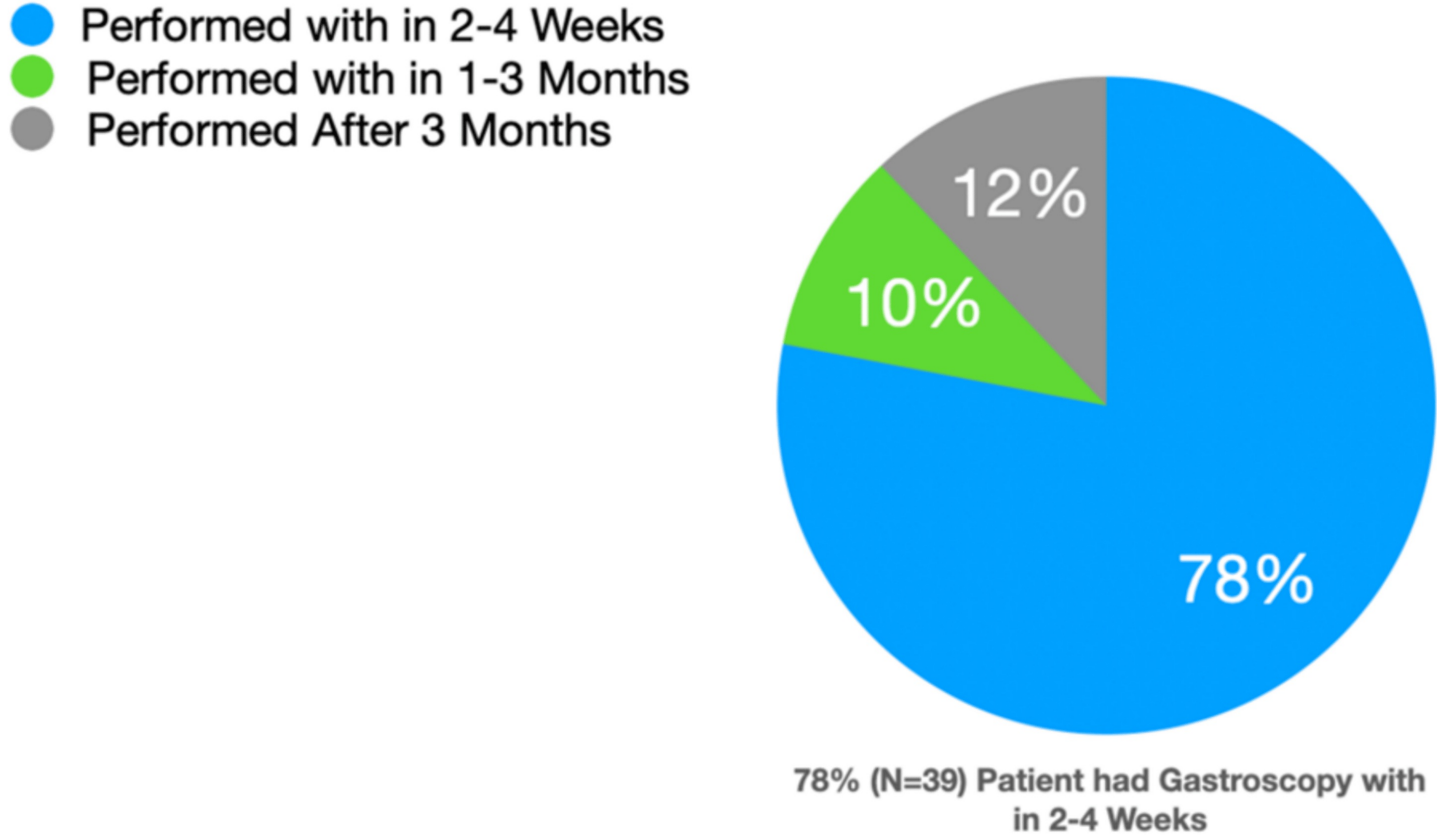
Time from referral to endoscopic examination

Time from referral to endoscopy: 78% of the cohort had gastroscopy within two to four weeks, 10% in four to 12 weeks and 12% after 12 weeks (Figure 1).

## Discussion

The results of our study demonstrate that a large majority of patients with incidental findings on their CT scans did not have oesophageal or gastric malignancies. Patients with malignancy showed a strong association with red-flag symptoms such as weight loss and iron deficiency anemia. Our results are similar to a study carried out by Castillo et al. which was conducted in the United States and included 126 patients with incidental EWT (IEWT) who underwent endoscopic examination. Their study revealed the endoscopic confirmation of cancer in only one patient [8]. Unlike our study though, Castillo et al. excluded all patients with symptoms suggestive of esophageal malignancy or known esophageal pathology.

However, Durmaz et al. detected much higher rates of esophageal cancer in their study in Turkey [3]. Their cohort included 144 patients with esophageal and GOJ thickness and found esophageal cancer in 53 patients (37%) in their cohort. It is also true however that they measured the wall thickness on CT and suggested that asymmetric wall thicknesses over 13.5 mm would be highly significant in terms of malignancy in tomographic examinations. Similarly, Tongdee et al. suggested a cut-off point of 10mm antral wall thickness on CT to differentiate between malignant and non-malignant conditions in the stomach [9]. In contrast, there were no set cut-off points for significant wall thickness in our study, and any GI wall thickness picked up on a CT scan was considered pathological and warranted further assessment with endoscopy. Another Turkish study that had a similarly high yield in picking up upper GI cancers was by Somuncu et al. who found 33 (29%) of their 88 patients with incidental upper GIWT had Gastric malignancies [10]. 

Whilst only one patient in our cohort had gastric cancer, it is also true that gastric wall thickness comprised a smaller proportion of patients in our study. Tellez-Avila et al. in their Mexican cohort found a much higher pick up of gastric malignancies with incidental GIWT on CT scans in the absence of any symptoms. In their research population, 19 patients had stomach wall thickness, and one patient had EWT, and they found stomach cancer in six patients on endoscopy [11]. Of interest in our study is the strong correlation of cancer findings with “red flag” symptoms in patients, particularly iron deficiency anemia, buttressing the importance of early endoscopy in this cohort of patients as established in many other studies [10-12].

Limitations in our studies include the fact that this was retrospective data of small sample size and that it covers only one single center in the UK. These factors may limit the generalizability of our findings. Retrospective data collection carries the risk of selection bias as there may have been an unintentional preference for certain groups of patients for referring to the endoscopic department for further evaluation. Specifically, patients with asymptomatic mild wall thickness may have not undergone endoscopic assessment. Additionally, a small sample size does not truly reflect the clinical significance of findings and increases the probability of type 2 error, where the true effect may be missed. Other limitations related to single-center studies as health care access, endoscopy referral pathway, radiologists' expertise in reporting GI wall thickness, and imaging techniques vary from center to center which further limits its generalizability. However, one of the key strengths of the present study is that this study comprehensively evaluated single-center UGIW thickness through both endoscopy and biopsy results. This dual analysis is valuable because gastroscopy offers direct visualization of mucosal pathologies and histological examination of biopsies helps to identify abnormal cells and differentiate malignant from benign conditions. Therefore, the probability of identifying cancer improves with a combination of both modalities. However, there are other factors as well that contribute to missing early esophageal cancer diagnosis on endoscopic examination including the endoscopist's expertise and inadequate biopsy sample [13]. Another important aspect of the present study is that, although it is a small single-center study, it provides evidence regarding the clinical significance of incidental upper GI wall thickness and lays the groundwork for future research in this under-explored area of medicine. Of course, for the development of more robust guidelines, there is an obvious need to carry out further multicenter large studies of a similar kind in other parts of the world. Other things to improve on in repeat studies would be an analysis of the exact measurements and criteria used by radiologists to define abnormal thickening of the GI wall for better accuracy. Overall, our study supports the referral of patients to an urgent cancer pathway in the presence of red-flag symptoms but in the absence of such symptoms for patients, particularly with EWT, a more detailed assessment with history and examination is required, and endoscopy should be conducted after a case-by-case review.

## Conclusions

This study shows that malignancies are much more common amongst patients who had mural upper GI wall thickness with red flag symptoms. Therefore, urgent endoscopy via the cancer pathway is recommended for this cohort of patients. However, the risk of malignancy is low among patients with IEWT and no red flag symptoms. We recommend that this group of patients need an individualized approach, and a detailed history and examination are advised to guide the need for further evaluation with endoscopy. However, further large studies are required to develop a standardized protocol in relation to investigations for people with IUGIW thickness on CT scans.
